# First-Time Magnetic Resonance Imaging Experience: A Descriptive Phenomenological Study with Implications for Nursing Preparation and Communication

**DOI:** 10.3390/nursrep16090294

**Published:** 2026-08-24

**Authors:** Sukanya Juntaree, Arissara Sukwatjanee, Duangduan Rattanamongkolgul

**Affiliations:** Faculty of Nursing, Srinakharinwirot University, Ongkharak 26120, Nakhon Nayok, Thailand; sukanyaj@g.swu.ac.th (S.J.); arissara@g.swu.ac.th (A.S.)

**Keywords:** magnetic resonance imaging, patient experience, qualitative research, descriptive phenomenology, anxiety, claustrophobia, nursing care, radiology nursing

## Abstract

**Background/Objectives:** Magnetic resonance imaging (MRI) is often supported by structured safety screening and preparation, yet first-time patients may still experience anxiety, claustrophobic distress, loss of control and non-completion. This study aimed to describe the experiences of adults undergoing MRI for the first time and to identify implications for nursing preparation, communication and diagnostic imaging service design. **Methods:** A descriptive phenomenological study informed by Husserlian philosophy was conducted in the radiology department of a university hospital in Thailand. Eleven adults who underwent first-time MRI were purposively recruited, including eight who completed and three who did not complete the examination; five participants were hospital personnel. Data were generated through in-depth semi-structured interviews, non-participant observation, audio recording and field notes, and were analysed using Colaizzi’s method. **Results:** Across the four themes, negotiation of control was identified as a descriptive cross-theme pattern grounded in participants’ accounts rather than as a separate interpretive construct. Participants entered MRI seeking diagnostic clarity, encountered a sensory and spatial environment that weakened control, attempted to regain control through coping and communication, and reflected on the support they would need for any future examination. Four interrelated themes were generated: becoming aware of bodily abnormality and accepting MRI; encountering the MRI environment; managing oneself inside the scanner; and reflecting after passing through the experience. **Conclusions:** The findings suggest that first-time MRI can be experienced as a nursing-relevant care encounter rather than a neutral technical event. Nursing preparation may need to move beyond routine instructions by addressing sensory realities, anxiety and claustrophobia, coping rehearsal, communication options and post-examination reflection. The effects of these strategies on completion and service outcomes require prospective evaluation.

## 1. Introduction

Magnetic resonance imaging (MRI) is widely used in contemporary health services because it provides detailed soft-tissue imaging without ionizing radiation and supports diagnosis, treatment planning and disease monitoring across multiple body systems [1,2]. Although MRI is commonly presented in clinical practice as safe, precise and routine, patients may encounter it as a high-technology care situation that requires removal of ordinary personal objects, positioning by staff, separation from accompanying relatives, immobility inside the scanner bore and tolerance of intense repetitive sound. From a person-centred perspective, these conditions matter because care is experienced through the body, emotions and relationships with professionals, not only through completion of a technical procedure [3,4]. Descriptive phenomenology is therefore useful for attending closely to how patients perceive and describe this situation while avoiding the imposition of a prior explanatory theory [5,6].

Patient anxiety in MRI is clinically important. Previous studies have shown that claustrophobia, anticipatory anxiety, unfamiliarity with the procedure, and difficulty remaining still can reduce cooperation, degrade image quality, prolong workflow, or lead to premature termination of the scan [7,8,9,10]. Qualitative work has also shown that patients may describe MRI as being “in another world”, a phrase that points to how the scanner environment can feel separate from ordinary clinical spaces and social interactions [7]. Recent reviews further indicate that patients’ MRI experiences are shaped by sensory overload, restricted movement, fear, waiting, perceived vulnerability, and the quality of communication with radiology staff [3,4,11].

Preparation interventions, including video demonstration, telephone contact, tailored information and relaxation strategies, may reduce pre-MRI anxiety or improve readiness [12,13]. MRI services also usually have structured preparation pathways, including safety screening, assessment of contraindications, explanation of the procedure, removal of metallic objects, positioning, and communication arrangements during scanning [14]. However, the existence of a formal preparation pathway does not necessarily mean that patients are psychologically or experientially prepared for the examination. Preparation that focuses only on instructions may miss the meanings patients are trying to construct: why their body needs investigation, what the machine will feel like, whether they can tolerate stillness, how they can ask for help, and what it would mean if they cannot complete the scan. Failure to complete or proceed with MRI is therefore not only an individual episode of anxiety; it can affect diagnostic timeliness, scanner workflow, staff workload, use of high-cost imaging resources and access for other patients waiting for MRI [7,8,9,10,15]. Patient-centred MRI preparation therefore requires attention to patients’ lived experience as well as to the professional service pathway.

In Thailand and similar middle-income health-service contexts, qualitative evidence about first-time MRI remains limited, particularly evidence that includes both patients who completed the scan and those who could not. At the study site, Radiology Information System and departmental service records indicated annual unsuccessful-examination rates of 7.04–8.9% during 2022–2025. For these service statistics, an unsuccessful examination referred to cancellation or termination after scanning had begun because the patient could not tolerate the procedure, including claustrophobic distress, anxiety or panic; no-shows and technical equipment failures were excluded. The present study focused specifically on patients’ accounts of completion and non-completion. Routine information and safety screening may be necessary but may not fully address fear, loss of control, claustrophobic distress and uncertainty during the scan. Deference to professionals, reluctance to interrupt a procedure, family-centred reassurance and spiritual coping were treated as contextually relevant possibilities to explore, not as assumed characteristics of Thai patients. Nurses assigned to diagnostic imaging units and MRI teams have a particularly important role because they may contribute to screening, preparation, positioning, reassurance, monitoring, communication, advocacy and post-examination care across the service pathway.

This study aimed to describe the experiences of adults undergoing MRI for the first time at a university hospital in Thailand and to identify implications for nursing preparation, communication and service design. The research question was: What are patients’ experiences before, during and after undergoing MRI for the first time?

## 2. Materials and Methods

### 2.1. Design

This qualitative study used descriptive phenomenology informed by Husserlian philosophy to describe the essential features of first-time MRI experiences from the perspectives of patients. The approach was appropriate because the study sought to describe what participants perceived, felt, imagined and did while moving through the MRI service pathway, while bracketing professional assumptions about what the procedure should mean to patients [5,6]. Terms such as ‘embodied’ refer here to participants’ concrete descriptions of breathing, bodily restriction, sound, spatial proximity and movement; they are used descriptively and do not signal a shift to hermeneutic or interpretive phenomenology. Likewise, ‘negotiation of control’ was retained only as a concise descriptive synthesis linking recurrent statements across the four themes, not as an a priori theory or an interpretation detached from the data. Reporting was guided by the Consolidated Criteria for Reporting Qualitative Research (COREQ) [16], and a completed item-by-item checklist is provided as Supplementary File S1.

### 2.2. Setting

The study was conducted in the radiology department of HRH Princess Maha Chakri Sirindhorn Medical Center, a university hospital in Nakhon Nayok, Thailand. The MRI pathway included appointment scheduling, screening for contraindications and precautions, pre-examination advice, preparation on the day of examination, changing clothes and removing metallic items, venous access when contrast was required, positioning on the MRI table, instructions about stillness and breath-holding when relevant, communication through the squeeze bulb/intercom, and post-examination care.

### 2.3. Participants and Recruitment

Participants were adults who had undergone MRI for the first time at the study site. Inclusion criteria were: first MRI at the radiology department, ability to provide consent independently, intact consciousness, ability to communicate in Thai, willingness to describe the experience, and no acute severe discomfort during interview. Exclusion criteria were inability to communicate sufficiently, physical or emotional unavailability for interview, or withdrawal during data collection. Purposive sampling was used to include direct experience of the phenomenon and variation in completion status. Recruitment and preliminary analysis proceeded concurrently. After each interview, the first author and supervisors reviewed the richness and relevance of the account, coverage of completion and non-completion, and whether additional interviews materially changed the developing meanings. Recruitment stopped at 11 when the accounts provided sufficient information power for the focused aim and subsequent interviews no longer added substantially different meanings, while still retaining variation in sex, age group, examination site and completion status [17].

Participants were six men and five women aged 34–66 years. Eight completed the MRI examination and three did not. Examination sites included the lumbar spine, brain, heart, knee and wrist. Five participants were hospital personnel and were recruited under the same eligibility and consent procedures as other participants. Two held support roles within the radiology department; none had a direct supervisory relationship with the interviewer, and the interviewer did not provide their direct MRI care on the examination day. Possible professional familiarity was documented reflexively and considered during interviewing and analysis. The interviewer emphasized voluntariness, confidentiality, the absence of consequences for care or employment, and the value of critical as well as positive accounts. To support transparency while reducing identifiability, individual-level characteristics are presented in Table 1 using participant codes and age groups.

### 2.4. Data Collection

Data were collected from December 2025 through January 2026, entirely within the renewed ethics-approval period. Participants were approached and scheduled before their MRI examination. After the examination was completed or terminated, the researcher met each participant and accompanied them to a nearby private interview room. All interviews began within one hour of the MRI encounter for both participants who completed and those who did not complete the examination; the precise start time varied according to each participant’s readiness. The first author conducted the in-depth semi-structured interviews in Thai. Interviews lasted approximately 45–60 min and were audio-recorded with permission. The interview guide used broad open-ended questions, including: ‘Could you tell me about your first MRI experience?’, ‘How did you feel before and during the scan?’, ‘What did you do to get through the examination?’, ‘How did you feel after the scan?’, and ‘What support would you want if you needed MRI again?’ Probes clarified feelings, thoughts, meanings, coping strategies and perceived needs. Non-participant observation was conducted with all participants during the interviews, focusing on facial expression, gaze, posture, emotion, tone of voice and language while experiences were recounted. Field notes were made during and immediately after each interview and were linked to the relevant transcript to contextualize and corroborate the verbal account; participants’ behaviour inside the closed scanner bore was not directly observed for research purposes.

### 2.5. Researcher Reflexivity and Bracketing

The first author was a nurse working in the radiology department and had more than nine years of experience with MRI service processes. This insider position was useful for understanding workflow and safety procedures, but it created risks of professional assumptions, prior professional familiarity and socially desirable responses, particularly among hospital personnel, including two participants in radiology support roles. The interviewer did not provide their direct MRI care on the examination day and had no direct supervisory authority over them. Recruitment followed the same eligibility and consent procedure for staff and non-staff participants, and consent emphasized confidentiality and that participation, withdrawal and critical comments would not affect care or employment. To reduce influence during interviews, the researcher used reflexive bracketing before, during and after interviews; conducted interviews privately outside the scanning area; used open questions rather than evaluative questions about service quality; avoided correcting or defending the department; invited both helpful and difficult experiences; and discussed emerging interpretations and possible insider bias with supervisors experienced in qualitative research.

### 2.6. Data Analysis

Interviews were transcribed verbatim and analysed using Colaizzi’s descriptive phenomenological method [6,18]. The first and third authors independently reviewed significant statements and formulated meanings; their coding and theme groupings were compared and resolved through discussion, and the second author reviewed the resulting structure for consistency. Analysis involved: (1) repeated reading of each transcript while listening to the recording; (2) extraction of significant statements relevant to first-time MRI; (3) formulation of meanings kept close to participants’ words; (4) clustering of meanings into subthemes and themes while checking divergence and negative cases; (5) development of an exhaustive description; (6) condensation into a descriptive cross-theme structure; and (7) return of the findings to participants for confirmation. For example, the statement ‘it felt like rehearsing death’ was retained as participant-described intrusive imagery and formulated as perceived threat within a confined sensory environment, rather than interpreted through an external symbolic theory. The four themes were distinguished analytically: ‘Encountering the MRI environment’ described sensory-spatial perceptions and immediate affective responses, whereas ‘Managing oneself inside the scanner’ described deliberate coping and communication actions. All analysis was conducted in Thai. Selected quotations were translated by the first author, then checked through forward-backward review by bilingual qualitative nursing researchers to preserve semantic, emotional and contextual equivalence; the Thai transcripts remained the reference text.

### 2.7. Trustworthiness

Trustworthiness was addressed using credibility, transferability, dependability and confirmability [19,20]. Credibility was enhanced through in-depth interviewing, non-participant observation during interviews, field notes and researcher triangulation. At the end of each interview, the interviewer summarized key points and invited correction or elaboration. After preliminary analysis, formulated meanings—concise statements derived from significant quotations while retaining their context—and the developing thematic structure were returned to all participants during follow-up contact, including by telephone, for confirmation or clarification. Additional information was incorporated into the analysis. The final descriptive structure was also checked through repeated return to transcripts, comparison across participants, the audit trail and co-author discussion. Transferability was supported by description of the setting, pathway, participants and service context. Dependability was supported by a documented analytic process and audit trail. Confirmability was strengthened through verbatim transcription, field notes, reflexive bracketing and participant quotations showing the link between data and description.

### 2.8. Ethical Considerations

The study was approved by the Human Research Ethics Committee of Srinakharinwirot University (approval number SWUEC-672103; initial approval 27 May 2024; renewed 14 October 2025). Participants received oral and written information about the study purpose, voluntary participation, confidentiality, audio recording, and the right to withdraw at any time without affecting care. Written informed consent was obtained before each interview. Names were replaced with participant codes and potentially identifying information was removed from quotations.

## 3. Results

The analysis generated four interrelated themes and nine subthemes. Across these themes, negotiation of control was identified as a descriptive cross-theme pattern grounded in recurrent participant statements: participants sought MRI to reduce diagnostic uncertainty, encountered a scanner environment in which control became fragile, used coping and communication to regain a sense of safety, and then reflected on support needed for any future examination. It was not treated as an additional interpretive theme or an a priori theoretical construct. Interview accounts provided the primary evidence. Observation during interviews and field notes added contextual evidence, such as tense posture, changes in tone and visible emotion while participants recounted distressing or relieving moments; these observations were used to corroborate or question the interpretation of the verbal accounts, not as direct observation of behaviour inside the scanner. The themes and subthemes are shown in Table 2, followed by a narrative account of each theme.

### 3.1. Becoming Aware of Bodily Abnormality and Accepting MRI

The first theme shows that the MRI experience began before participants entered the scanner. It began with bodily symptoms that disrupted daily life and raised uncertainty about diagnosis and treatment. Acceptance of MRI was therefore not passive compliance; it was tied to hope that the scan would make illness understandable and guide further care.

Bodily symptoms and diagnostic uncertainty made MRI meaningful before the procedure began. The scan was accepted because participants believed it could make their illness visible and help clinicians decide on treatment.

#### 3.1.1. Perceiving Bodily Symptoms

Participants described symptoms such as headache, back pain, hip pain, knee pain, chest discomfort, hearing problems, numbness, leg weakness, and difficulty walking. These symptoms disrupted daily life and work and led participants to seek medical care. When symptoms persisted despite initial treatment, MRI was perceived as a necessary next step.

“My back had already been X-rayed, but the doctor said the bones overlapped. I needed CT and MRI so they could decide whether I needed surgery, injections, or physiotherapy.”

(P1)

“The pain came back when I worked and lifted things. This time it was too much. The doctor said I should have MRI.”

(P2)

#### 3.1.2. Accepting and Preparing for MRI

Most participants accepted the examination because they trusted the physician’s recommendation and believed MRI could help clarify the diagnosis. Preparation involved appointment scheduling, blood tests when needed, screening for contraindications, changing clothes, removing metallic items, and receiving instructions from staff. These steps increased procedural understanding but did not always remove anxiety about the real situation inside the scanner.

“The nurse asked whether I was afraid of narrow spaces, whether I had ever had MRI, and whether I had any disease. They told me that if it felt hot or pulled, I should squeeze the bulb.”

(P3)

“They told me which medicine to stop and to have blood taken before the scan. I just followed the steps.”

(P4)

### 3.2. Encountering the MRI Environment

The second theme captured participants’ sensory-spatial perceptions and immediate feelings as they moved from knowing about MRI to being physically inside the MRI environment. It concerns what the room, machine, bore, body positioning, immobility and acoustic noise felt like; the subsequent theme, ‘Managing oneself inside the scanner’, concerns what participants deliberately did in response. For some, the environment was tolerable; for others, it rapidly converted uncertainty into fear and perceived loss of control.

#### 3.2.1. Perceiving the Examination Environment

Participants remembered the room as white, large, cold, and dominated by a tunnel-like machine. The scanner bore was often described as narrow, close to the face or body, and limiting. The sound was one of the most memorable features, with participants comparing it to knocking, pounding on walls, a train, a car horn, loud speakers, or chopping meat. These descriptions show that the MRI environment was not a neutral technical space but a sensory environment that participants actively interpreted.

“The table moved into the tunnel. I looked up and saw the low white ceiling. It was only about fifteen centimetres away. I felt excited because I had never been in that place before.”

(P1)

“The noise was very loud. It was like someone banging on the walls all around the room. It made me think, if it collapsed, how would I get out?”

(P11)

“It wasn’t frightening the way people say. They told me it would take about 45 min, but for me that was nothing unusual. The sound that people say is loud—for me it was like listening to music. I just lay there and listened calmly. There was nothing to be afraid of.”

(P10)

#### 3.2.2. Feelings During the Examination

Participants’ feelings ranged from excitement, tightness in the chest, breathlessness, claustrophobic fear, sweating, restlessness and panic to calmness and acceptance. Three participants could not complete the examination. Their accounts suggest that non-completion was not simple refusal; it occurred when the body and mind could no longer tolerate the combination of narrow space, body restriction, sound, duration and perceived inability to escape.

“I lay down and could not breathe comfortably. It felt tight and suffocating, as if I would die. I wanted to do it, but my body could not.”

(P4)

“It was so narrow. I had to keep my arm raised and stay still for a long time. The space felt as if it forced my body and my mind.”

(P6)

“I stayed still, closed my eyes, and did what the doctor said. For me it was not as frightening as people say.”

(P7)

#### 3.2.3. Intrusive Imagery During the Examination

Several participants described intrusive imagery associated with the scanner. Three explicitly described distressing comparisons with rehearsing death, a coffin or drowning, while a fourth described a negative case in which anticipated crematorium imagery did not materialize after entering the scanner. These images were participants’ own comparisons and are presented descriptively rather than as symbolic interpretations imposed by the researchers. The negative case shows that first-time MRI is not universally frightening and that preparation should be individualized rather than based on assumptions.

“Honestly, it felt like rehearsing death. The tunnel was narrow and I could not breathe well. I thought a coffin might be only a little bigger than this.”

(P1)

“It felt like being in a coffin. I thought that if I stayed there, I would surely die. I could not stay.”

(P11)

“It was like drowning. I felt tight in the chest and could not breathe.”

(P6)

“Honestly, there was nothing scary about it at all. Before I went in, I imagined all kinds of things—that it might feel like lying in a crematorium. But once I was actually inside, it wasn’t frightening the way I had imagined. It was calm. I just lay still, and there was nothing there.”

(P9)

### 3.3. Managing Oneself Inside the Scanner

In contrast to the preceding theme’s focus on perceptions and immediate feelings, this theme describes participants’ deliberate responses while inside the scanner. Although they could not control the scanner bore, noise or duration, they attempted to regulate breathing, attention, thoughts, prayer, stillness and communication with staff. These actions were described as ways of restoring a workable sense of safety within an environment that limited bodily freedom.

#### 3.3.1. Distraction and Attentional Control

Participants used diverse strategies to distract themselves from fear and discomfort, including closing their eyes, breathing slowly, counting, meditation, chanting, prayer, recalling God or sacred figures, and repeatedly telling themselves to endure for the sake of diagnosis and treatment. These strategies helped some participants create a mental anchor in a situation of limited control.

“I closed my eyes. I breathed in Budd and breathed out Dho, slowly and deeply. It helped me stay with myself and not focus on the surrounding noise.”

(P1) [“Budd” and “Dho” are the two syllables of “Buddho”, a tradi-tional Thai Buddhist meditation mantra silently recited on the in-breath and out-breath.]

“I told myself not to be afraid. I had to do this for treatment. I tried to keep my mind calm and still.”

(P6)

“I counted from one to one hundred and started again. Sometimes I prayed. It reduced the worry and helped me get through it.”

(P3)

“When I lay on the table and it slid into the machine, I saw the tunnel—narrow, white. At first I felt tight, but I told myself to stay still and calm and just accept it. I didn’t need to get excited, just act normal. When the doctor said to breathe in, breathe out, hold my breath, I followed along. From more than twenty years of diving to catch fish, where I had to control my breathing underwater for long periods, I already knew how to do this, and it helped me get through it.”

(P5)

#### 3.3.2. Communication During the Examination

The squeeze bulb was an important communication channel. Participants viewed it not only as an emergency device but also as proof that they were not completely alone inside the machine. For those who became unable to tolerate the scan, squeezing the bulb was the action that ended the examination and restored a sense of safety.

“I tried to lie down, but I could not. I wanted to do the scan, but I had to squeeze the bulb and ask them to take me out.”

(P4)

“I pressed the alarm. I could not stay. I told them I was afraid and felt as if I would faint.”

(P11)

### 3.4. Reflecting After Passing Through the Experience

After the examination, participants reflected on what they had endured, what they had learned about themselves, and what support they would need if MRI were required again. The end of the scan was often experienced as release, but not always as closure. For those with intense fear or non-completion, the memory shaped future expectations and their preferred form of support.

#### 3.4.1. Feelings After the Examination

Most participants described relief after leaving the scanner. Relief was linked to regaining bodily freedom: breathing fully, moving again, and leaving the narrow space. Some felt proud that they had completed the scan, while others felt relieved yet reluctant to undergo MRI again unless medically necessary.

“When I came out of the machine, I felt open and relieved. My body could breathe fully again and move again.”

(P1)

“It was like being released from confinement. Outside, I could breathe much better.”

(P11)

“If I could choose, I would avoid it. But if it is really necessary, I have to do what the doctor says.”

(P6)

#### 3.4.2. Needs for Future Support

Participants who experienced severe fear expressed a need for additional options if they required MRI again. These included having a trusted relative nearby, receiving stronger reassurance, or being sedated or anaesthetized when clinically appropriate. These needs reflect participants’ desire for emotional safety, a reliable way to stop the examination, and a more tolerable examination process.

“Next time, I would want a relative in the room, or I would want the doctor to help me sleep through it.”

(P8)

“If I have to do it again, I want anaesthesia. If I am awake, I do not think I can stay.”

(P11)

## 4. Discussion

This study describes first-time MRI across diagnostic hope, sensory intensity, restricted control, self-management and post-examination reflection. Its main original contribution is the comparison embedded within a single phenomenological sample of patients who completed MRI and those who could not. Accounts of non-completion showed that stopping was not simple refusal but the point at which bodily distress, intrusive imagery, spatial restriction and perceived inability to escape became intolerable. The study also contributes culturally situated descriptions of participant-generated coping, while avoiding the assumption that these strategies characterize all Thai patients. Together, these findings identify a preparation pathway–patient experience gap: structured safety screening and procedural instructions do not necessarily prepare patients for the sensory, emotional and relational demands of the scan.

The first theme shows that the MRI experience began with bodily symptoms and the search for answers. Participants’ acceptance of MRI was grounded in hope that the examination would explain persistent symptoms and guide treatment, consistent with evidence that imaging experiences are shaped by illness uncertainty, expectations about diagnostic value and fear of what the examination may reveal [3,4,21,22]. This finding also foregrounds vulnerability and trust: patients accepted temporary dependence on staff and technological processes because they expected diagnostic benefit. For nurses, person-centred preparation therefore includes eliciting what the patient fears, hopes to learn and understands about the scan, while making space for questions rather than treating compliance as evidence of readiness.

The second theme highlights sensory and spatial features of MRI as central to experience. The white room, tunnel-like machine, proximity to the face and body, and repetitive loud noise were remembered vividly. These features match previous descriptions of MRI as a potentially stressful environment that can provoke anxiety, claustrophobia, and premature termination [7,8,9,10]. However, this study also shows that the same environment did not affect all patients in the same way. Some interpreted the sound as frightening or overwhelming; others regarded it as ordinary or even like music. This variation supports the need for individualized screening rather than assuming that all first-time patients will either tolerate or fear MRI in the same way.

Intrusive imagery was a clinically important participant-described feature. Three participants explicitly compared the scanner with rehearsing death, a coffin or drowning, while a fourth described anticipated crematorium imagery that subsided after entering the scanner. Previous reviews have described claustrophobia, feeling trapped and fear as recurrent components of MRI experience [3,4,11]. The present findings add descriptive detail about the images through which some participants communicated bodily threat. These accounts should not be read as universal symbols or as researcher-imposed interpretation. They suggest that staff may ask about feared sensations or images in simple, non-judgmental language and explain what patients can do if distress occurs.

The third theme demonstrates that patients actively managed themselves in the scanner through breathing, counting, meditation, prayer and internal self-talk. These strategies are consistent with evidence that preparation and coping techniques may reduce MRI-related anxiety and support cooperation [12,13]. In this Thai sample, Buddhist breathing phrases, prayer and remembrance of sacred or religious figures were participant-generated strategies used by some individuals. They should not be generalized to all Thai patients or transferred to other cultural groups as prescribed techniques. Nurses and radiographers can instead invite each patient to identify a personally acceptable calming strategy and, where appropriate, rehearse it before entering the room. This approach supports agency and culturally responsive care without stereotyping.

Communication with staff also emerged as a relational safety mechanism. The squeeze bulb was not simply a device for alerting staff; participants described it as evidence that help remained available despite physical separation. This finding links perceived control with trust, vulnerability and the therapeutic relationship. Prior studies have emphasized radiology staff communication, reassurance and patient-centred interaction [11,23]. The accounts suggest that staff should explain and demonstrate the squeeze bulb, confirm that monitoring continues and explicitly state that asking for help is acceptable. Whether these practices reduce anxiety or non-completion requires prospective evaluation.

The final theme shows that the experience continued after the scan. Relief was common, but some participants remained reluctant to undergo MRI again. A short post-scan conversation, especially after severe fear or termination, may help identify reasons for distress, document preferred support and inform a future imaging plan. Options generated from these findings and prior literature include enhanced preparation, audiovisual explanation, accompaniment when safe and feasible, anxiety-management coaching, or medical sedation pathways when clinically indicated [10,12,13]. These are practice implications, not interventions tested by the present study; their effects on patient outcomes, repeat appointments, workflow and access should be evaluated prospectively.

### 4.1. Implications for Nursing Preparation and Service Design

The findings generate practical nursing and service-design considerations. Radiology nurses, nurses assigned to diagnostic imaging units and MRI teams may screen first-time service users for claustrophobia, anxiety, breathlessness, difficulty lying flat, pain and concerns about the scanner environment; provide sensory information about the bore, noise, temperature, duration and immobility; demonstrate the squeeze bulb or call system; and develop individualized plans for patients reporting severe fear or intrusive imagery. A brief post-examination conversation may also identify unresolved distress if repeat MRI is required. These strategies are hypotheses for person-centred practice derived from qualitative accounts. Their feasibility and effects on anxiety, completion and resource use were not evaluated here and require prospective study.

### 4.2. Strengths and Limitations

A strength of this study is the inclusion of both patients who completed MRI and those who did not, allowing the analysis to capture a wider range of first-time MRI experiences. In-depth interviews, field notes, reflexive bracketing, member checking and supervisory debriefing strengthened the trustworthiness of the findings. The study also provides culturally situated evidence from Thailand, where qualitative research on first-time MRI experiences remains limited.

Several limitations should be noted. The study was conducted in a single university hospital, and findings may not represent other hospitals, scanner types or service systems. Five participants were hospital personnel; professional familiarity with health services and possible prior familiarity with the interviewer or setting may have influenced what they noticed and their willingness to speak critically. The interviewer’s insider position in the radiology department may also have shaped recruitment, questioning and interpretation despite bracketing, neutral prompts, audit-trail documentation and co-author debriefing. Most participants were Buddhist, and the participant-generated religious or spiritual strategies should not be generalized to all Thai patients or other cultural and faith groups. Although all interviews began within one hour of the MRI encounter, participants’ accounts remain retrospective descriptions rather than direct observation of experiences inside the scanner. Observation was limited to accessible phases outside the closed bore. These limitations should be considered when transferring the findings.

## 5. Conclusions

In this single-centre descriptive phenomenological sample, first-time MRI was described as an experience that began with symptoms and diagnostic uncertainty, intensified in the sensory and spatial environment of the scanner, prompted active self-management under restricted control and continued into post-examination reflection. The findings support consideration of individualized sensory, emotional and culturally responsive nursing preparation, including assessment of anxiety and claustrophobia, rehearsal of patient-chosen coping strategies, clear communication options and follow-up after distress. These implications are grounded in participants’ accounts, including those who did not complete MRI; they should not be interpreted as evidence that the proposed strategies improve completion or resource use. Prospective studies are needed to evaluate feasibility and outcomes across diverse imaging settings.

## Figures and Tables

**Table 1:** Participant characteristics.

Participant	Age Group	Sex	MRI Site	Completed MRI
P1	50s	Male	Lumbar spine	Yes
P2	30s	Female	Lumbar spine	Yes
P3	40s	Male	Left knee	Yes
P4	50s	Male	Heart	No
P5	40s	Male	Heart	Yes
P6	40s	Female	Wrist	Yes
P7	60s	Male	Brain	Yes
P8	30s	Female	Brain	No
P9	50s	Female	Knee	Yes
P10	60s	Male	Brain	Yes
P11	40s	Female	Lumbar spine	No

Note. Age groups are reported broadly to reduce identifiability. Participant codes replace pseudonyms used in the original Thai transcripts.

**Table 2:** Themes and subthemes.

Themes	Subthemes
1. Becoming aware of bodily abnormality and accepting MRI	1.1 Perceiving bodily symptoms 1.2 Accepting and preparing for MRI
2. Encountering the MRI environment	2.1 Perceiving the examination environment 2.2 Feelings during the examination 2.3 Intrusive imagery during the examination
3. Managing oneself inside the scanner	3.1 Distraction and attentional control 3.2 Communication during the examination
4. Reflecting after passing through the experience	4.1 Feelings after the examination 4.2 Needs for future support

## Data Availability

The data presented in this study are available on request from the corresponding author due to privacy and ethical restrictions. The data are not publicly available because they contain potentially identifiable qualitative interview data. Anonymized quotations supporting the findings are included in the article.

## Supplementary Materials

The following supporting information can be downloaded at: https://www.mdpi.com/article/10.3390/nursrep16090294/s1, Supplementary File S1: Completed COREQ checklist mapping for this qualitative interview study.

## Public Involvement Statement

No public involvement in any aspect of this research.

## Guidelines and Standards Statement

This manuscript was drafted against the Consolidated Criteria for Reporting Qualitative Research (COREQ) for qualitative interview research.

## Abbreviation

MRI	Magnetic resonance imaging

## References

[1] Edelman R.R. (2014). The history of MR imaging as seen through the pages of Radiology. Radiology.

[2] Smith-Bindman R., Miglioretti D.L., Larson E.B. (2008). Rising use of diagnostic medical imaging in a large integrated health system. Health Aff..

[3] Munn Z., Jordan Z. (2011). The patient experience of high technology medical imaging: A systematic review of the qualitative evidence. JBI Evid. Synth..

[4] Madl J.E., Nieto Alvarez I., Amft O., Rohleder N., Becker L. (2024). The psychological, physiological, and behavioral responses of patients to magnetic resonance imaging (MRI): A systematic review and meta-analysis. J. Magn. Reson. Imaging.

[5] Husserl E. (1970). The Crisis of European Sciences and Transcendental Phenomenology: An Introduction to Phenomenological Philosophy.

[6] Colaizzi P., Valle R.S., King M. (1978). Psychological research as the phenomenologist views it. Existential-Phenomenological Alternatives for Psychology.

[7] Törnqvist E., Månsson Å., Larsson E.M., Hallström I. (2006). It is like being in another world: Patients’ lived experience of magnetic resonance imaging. J. Clin. Nurs..

[8] Tazegul G., Etcioglu E., Yildiz F., Yildiz R., Tuney D. (2015). Can MRI related patient anxiety be prevented?. Magn. Reson. Imaging.

[9] Eshed I., Althoff C.E., Hamm B., Hermann K.G.A. (2007). Claustrophobia and premature termination of magnetic resonance imaging examinations. J. Magn. Reson. Imaging.

[10] Murphy K.J., Brunberg J.A. (1997). Adult claustrophobia, anxiety and sedation in MRI. Magn. Reson. Imaging.

[11] Lawal O., Regelous P., Omiyi D. (2023). Supporting claustrophobic patients during magnetic resonance imaging examination: The patient perspective. Radiography.

[12] Tugwell J., Goulden N., Mullins P. (2018). Alleviating anxiety in patients prior to MRI: A pilot single-centre single-blinded randomised controlled trial to compare video demonstration or telephone conversation with a radiographer versus routine intervention. Radiography.

[13] Hamd Z.Y., Alorainy A.I., Alrujaee L.A., Alshdayed M.Y., Wdaani A.M., Alsubaie A.S., Binjardan L.A., Kariri S.S., Alaskari R.A., Alsaeed M.M. (2023). How different preparation techniques affect MRI-induced anxiety of MRI patients: A preliminary study. Brain Sci..

[14] Pedrosa I., Altman D.A., Dillman J.R., Hoff M.N., McKinney A.M., Reeder S.B., Rogg J.M., Stafford R.J., Webb J.A., Hernandez D.L. (2025). American College of Radiology Manual on MR Safety: 2024 update and revisions. Radiology.

[15] AlRowaili M.O., Ahmed A.E., Areabi H.A. (2016). Factors associated with no-shows and rescheduling MRI appointments. BMC Health Serv. Res..

[16] Tong A., Sainsbury P., Craig J. (2007). Consolidated criteria for reporting qualitative research (COREQ): A 32-item checklist for interviews and focus groups. Int. J. Qual. Health Care.

[17] Malterud K., Siersma V.D., Guassora A.D. (2016). Sample size in qualitative interview studies: Guided by information power. Qual. Health Res..

[18] Morrow R., Rodriguez A., King N. (2015). Colaizzi’s descriptive phenomenological method. Psychologist.

[19] Guba E.G., Lincoln Y.S., Denzin N.K., Lincoln Y.S. (1994). Competing paradigms in qualitative research. Handbook of Qualitative Research.

[20] Korstjens I., Moser A. (2018). Series: Practical guidance to qualitative research. Part 4: Trustworthiness and publishing. Eur. J. Gen. Pract..

[21] Busacchio D., Mazzocco K., Gandini S., Pricolo P., Masiero M., Summers P.E., Pravettoni G., Petralia G. (2021). Preliminary observations regarding the expectations, acceptability and satisfaction of whole-body MRI in self-referring asymptomatic subjects. Br. J. Radiol..

[22] Nieto-Alvarez I., Madl J., Becker L., Amft O. (2025). Patients’ experience to MRI examinations: A systematic qualitative review with meta-synthesis. J. Magn. Reson. Imaging.

[23] Ajam A.A., Xing B., Siddiqui A., Yu J.S., Nguyen X.V. (2021). Patient satisfaction in outpatient radiology: Effects of modality and patient demographic characteristics. J. Patient Exp..

