# Supplementary material for: First-Time Magnetic Resonance Imaging Experience: A Descriptive Phenomenological Study with Implications for Nursing Preparation and Communication
Source: Nurs Rep. 2026 Aug 24;16(9):294. doi: 10.3390/nursrep16090294 (PMC13610125; doi:10.3390/nursrep16090294)
Supplement: Supplementary file 1 [file nursrep-16-00294-s001.zip › nursrep-4459437-supplementary.pdf]

Supplementary File S1. COREQ Checklist Mapping for the Qualitative Interview Study

First-Time Magnetic Resonance Imaging as an Embodied Care Encounter: A Descriptive Phenomenological Study with Implications for Nursing Preparation and Communication

This supplementary file maps the reporting of this descriptive phenomenological interview study against the Consolidated Criteria for Reporting Qualitative Research (COREQ) 32-item checklist. The mapping refers to the submitted manuscript sections and, where relevant, to details included in this supplementary file for transparency.

COREQ domain	Item	COREQ criterion	Reported in this study / manuscript location
Domain 1: Research team and reflexivity	1	Interviewer/facilitator	Reported in Materials and Methods: Data Collection and Researcher Reflexivity and Bracketing. The first author conducted the interviews.
Domain 1: Research team and reflexivity	2	Credentials	Reported in author information and manuscript context. The first author was a nurse working in the radiology department; supervisors were nursing academics experienced in qualitative research.
Domain 1: Research team and reflexivity	3	Occupation	Reported in Researcher Reflexivity and Bracketing. The first author was a nurse in the radiology department with more than nine years of MRI service experience.
Domain 1: Research team and reflexivity	4	Gender	Not emphasized in the main manuscript because the analysis focused on the interviewer's professional insider position rather than gender. This was not considered analytically central to the phenomenon.
Domain 1: Research team and reflexivity	5	Experience and training	Reported in Researcher Reflexivity and Bracketing and Trustworthiness. The interviewer had professional MRI service experience; supervisors had qualitative research expertise and supported supervisory debriefing.
Domain 1: Research team and reflexivity	6	Relationship established	Reported in Data Collection and Ethical Considerations. Participants were approached for voluntary participation, provided informed consent, and were interviewed privately after receiving study information.
Domain 1: Research team and reflexivity	7	Participant knowledge of the interviewer	Reported in Researcher Reflexivity and Bracketing. The manuscript acknowledges the interviewer's insider role in the radiology department and the possibility of socially desirable responses.
Domain 1: Research team and reflexivity	8	Interviewer characteristics	Reported in Researcher Reflexivity and Bracketing. The manuscript describes the interviewer's clinical role, insider position, assumptions, risks, and reflexive strategies.
Domain 2: Study design	9	Methodological orientation and theory	Reported in Materials and Methods: Design. The study used descriptive phenomenology informed by Husserlian philosophy and Colaizzi's method.
Domain 2: Study design	10	Sampling	Reported in Participants and Recruitment. Purposive sampling was used to recruit adults with direct first-time MRI experience, including those who completed and did not complete the examination.
Domain 2: Study design	11	Method of approach	Reported in Participants and Recruitment and Ethical Considerations. Eligible adults were recruited at the study site and provided written informed consent before interview.
Domain 2: Study design	12	Sample size	Reported in Participants and Recruitment. Eleven adults participated, including eight who completed and three who did not complete MRI.
Domain 2: Study design	13	Non-participation	No withdrawal during data collection was reported in the final dataset. The manuscript states withdrawal was permitted at any time without affecting care.
Domain 2: Study design	14	Setting of data collection	Reported in Setting and Data Collection. Interviews were conducted in a private room associated with the radiology service context of a university hospital in Thailand.
Domain 2: Study design	15	Presence of non-participants	Reported in Data Collection as private interviews. No non-participant was present during interviews.
Domain 2: Study design	16	Description of sample	Reported in Participants and Recruitment and Table 1. Participant sex, age group, MRI site and completion status are summarized with participant codes to reduce identifiability.
Domain 2: Study design	17	Interview guide	Reported in Data Collection. Broad open-ended questions and probes are provided in the manuscript.
Domain 2: Study design	18	Repeat interviews	The study relied primarily on one in-depth interview per participant. Follow-up contact was used only when clarification of formulated meanings was needed.
Domain 2: Study design	19	Audio/visual recording	Reported in Data Collection and Ethical Considerations. Interviews were audio-recorded with participants' permission.
Domain 2: Study design	20	Field notes	Reported in Data Collection and Trustworthiness. Field notes documented context, non-verbal responses, tone of voice and analytic reflections.
Domain 2: Study design	21	Duration	Reported in Data Collection. Interviews lasted approximately 45–60 minutes.
Domain 2: Study design	22	Data saturation	Reported in Participants and Recruitment. Recruitment continued until interviews yielded rich, detailed accounts and no substantially new meanings were identified relative to the study aim; sample size was guided by information power.
Domain 2: Study design	23	Transcripts returned	Full transcripts were not returned to participants. Member checking was conducted by summarizing key points at the end of interviews and, when clarification was needed, by follow-up contact to confirm formulated meanings.
Domain 3: Analysis and findings	24	Number of data coders	Reported in Data Analysis and Author Contributions. The first author led formal analysis with supervisory review and validation by the co-authors.

COREQ domain	Item	COREQ criterion	Reported in this study / manuscript location
Domain 3: Analysis and findings	25	Description of coding tree	Reported in Data Analysis and Results. Significant statements were identified, meanings were formulated, and similar meanings were clustered into nine subthemes and four themes; Table 2 presents the thematic structure.
Domain 3: Analysis and findings	26	Derivation of themes	Reported in Data Analysis and Results. Themes were derived inductively from participant accounts using Colaizzi's descriptive phenomenological method.
Domain 3: Analysis and findings	27	Software	No qualitative data analysis software was reported. Analysis was conducted through repeated reading, statement extraction, formulation of meanings and clustering using Colaizzi's method.
Domain 3: Analysis and findings	28	Participant checking	Reported in Trustworthiness. Key points were summarized with participants at the end of interviews and follow-up contact was used when clarification was needed.
Domain 3: Analysis and findings	29	Quotations presented	Reported throughout Results. Participant quotations are presented with participant codes (P1–P11).
Domain 3: Analysis and findings	30	Data and findings consistent	Reported throughout Results. Each theme is supported by narrative interpretation and participant quotations.
Domain 3: Analysis and findings	31	Clarity of major themes	Reported in Results and Table 2. Four major themes are clearly named and described.
Domain 3: Analysis and findings	32	Clarity of minor themes	Reported in Results and Table 2. Nine subthemes are listed and elaborated in the narrative findings.

Note. COREQ = Consolidated Criteria for Reporting Qualitative Research; MRI = magnetic resonance imaging. This checklist is provided as supplementary reporting support and does not replace the main manuscript narrative.
